# Precision Vinification Without Added Sulphur Dioxide: Real-Time Gas Monitoring Across Multiple Vintages

**DOI:** 10.3390/foods15030563

**Published:** 2026-02-05

**Authors:** Nicola Mercanti, Monica Macaluso, Andrea Marianelli, Ilaria Mannucci, Bruno Casu, Fabrizio Palla, Piero Giorgio Verdini, Massimo Fedel, Angela Zinnai

**Affiliations:** 1 Department of Agriculture, Food and Environment, University of Pisa, Via del Borghetto 80, 56124 Pisa, Italy; nicola.mercanti@phd.unipi.it (N.M.); monica.macaluso@unipi.it (M.M.); i.mannucci3@studenti.unipi.it (I.M.); angela.zinnai@unipi.it (A.Z.); 2 INFN Pisa Section, Largo Bruno Pontecorvo 3, 56127 Pisa, Italy; bruno.casu@cern.ch (B.C.); fabrizio.palla@cern.ch (F.P.); 3 European Organization for Nuclear Research, Espl. des Particules 1, 1211 Meyrin, Switzerland; piero.giorgio.verdini@cern.ch; 4 IFN CNR (Istituto di Fotonica e Nanotecnologie, Consiglio Nazionale delle Ricerche), Via Trasea 7, 35131 Padova, Italy; massimo.fedel@pd.ifn.cnr.it; 5 Interdepartmental Research Centre “Nutraceuticals and Food for Health”, University of Pisa, Via del Borghetto 80, 56124 Pisa, Italy

**Keywords:** sulphite-free wine, smart tank, sustainable oenology, no-added-SO_2_ protocol, Sangiovese, additives reduction

## Abstract

The reduction or elimination of sulphur dioxide (SO_2_) in winemaking represents a major technological and sustainability challenge due to the central antimicrobial and antioxidant roles of this additive. This study evaluated the technological feasibility and chemical stability of a no-added-SO_2_ vinification protocol applied under controlled winery conditions over four consecutive vintages, compared with a conventional sulphite-based protocol. The no-added-SO_2_ protocol integrated closed-circuit operations, controlled inert gas management, temperature-regulated fermentation, strict hygiene practices, the addition of grape seed extracts as alternative antioxidant agents, and real-time monitoring of CO_2_ production and O_2_ availability via a smart tank. Across all vintages, wines produced using the no-added-SO_2_ protocol showed regular alcoholic and malolactic fermentations and volatile acidity values consistently below the sensory perception threshold (1.2 g/L). Total SO_2_ levels ranged between 0.3 and 86 mg/L and free SO_2_ ranged between 0.4 and 16 mg/L, attributable exclusively to endogenous yeast production. Multivariate analysis confirmed that vintage was the dominant factor affecting most compositional parameters, particularly phenolic and anthocyanin profiles, whereas sulphur dioxide management represented a secondary but clearly identifiable source of variability. These findings indicate that sulphur dioxide-free vinification is technically feasible when supported by precise process control and continuous real-time monitoring. Rather than a universal replacement for conventional sulphite management, the no-added-SO_2_ protocol should be regarded as a complementary and technologically contingent tool for sustainable SO_2_ reduction within a precision oenology framework.

## 1. Introduction

In the last few years, climate change has taken a central role in analyses of the evolution of agricultural systems, influencing not only the physical and chemical parameters of production but also the economic and social dynamics associated with it [1,2,3]. According to a study conducted in Germany [4], extreme weather events such as droughts, abnormal rainfall, and heat waves have a direct impact on yields and the availability of agricultural resources, highlighting the importance of adaptive responses implemented by farmers and markets: from changes in cultivated areas to the intensification of production practices and price changes. The wine sector is particularly sensitive to these changes. The increase in average temperatures, irregular rainfall, and the frequency of extreme weather events significantly alter the phenology of vines [5,6,7]. The temperature and water variations induced by climate change are accelerating the ripening process of grapes, characterised by faster sugar accumulation, decreased acidity, and alterations in the aromatic profile, which translate into an overall earlier harvest [8]. Furthermore, extreme climatic conditions can inhibit the metabolism of the vine, reducing the accumulation of metabolites essential for aroma and colour [9]. The fermentation process is also affected: musts with a high sugar content cause stress in yeasts, with increased formation of undesirable products (such as acetic acid) and changes in the microbial balance, with potential risks of organoleptic deterioration [10].

Climate change is increasingly affecting viticulture through higher temperatures, altered rainfall patterns, and more frequent extreme events, which can influence grape composition, fermentation dynamics, and technological risk profiles [11,12,13].

In particular, accelerated sugar accumulation, reduced acidity, and altered microbial balance in musts may increase fermentation stress and the formation of undesirable by-products, thereby raising the risks of microbiological and oxidative instability in wine production [14,15,16,17].

This is the context in which the issue of sulphur dioxide occurs. Sulphur dioxide (SO_2_) plays a central technological role in winemaking due to its antimicrobial and antioxidant properties, contributing to the control of spoilage microorganisms, oxygen scavenging, and the binding of carbonyl compounds such as acetaldehyde. However, it is increasingly under scrutiny due to its possible negative effects on health [18,19,20,21,22,23]. The growing popularity of organic wines and products labelled “no added sulphites” reflects increasing consumer awareness and a real need to identify effective alternative strategies for protecting wine without compromising its sensory quality and stability over time [19,24]. Detailed analysis of seasonal meteorological variables, such as average temperatures and rainfall, is crucial to understanding the influence of environmental conditions on grape ripening, the fermentation process and, ultimately, the organoleptic characteristics of the wine produced [25,26,27,28]. The collection and interpretation of this data allows for evaluation of the adaptation of the alternative technique in relation to the climatic variations observed, providing a scientific basis for optimising winemaking practices and ensuring product quality over time [15]. Extreme and unpredictable climatic conditions have characterised recent wine-growing seasons, increasing the risk of microbiological and oxidative instability in must and wine. This study evaluated the use of an intelligent winemaking system and a MASAF (Italian Ministry of Agriculture, Food Sovereignty and Forestry) licensed operating protocol as a possible alternative to the addition of sulphur dioxide. Through the automated control of fermentation parameters and the analysis of the chemical–physical and aromatic characteristics of the wines obtained, the study aims to verify whether technological innovation can be an effective tool for combining quality, safety, and sustainability in contemporary wine production. The validation of the proposed innovation required the use of advanced technologies to support traditional chemical–analytical analyses, with the aim of monitoring gas conditions during the winemaking phase.

Despite the growing literature on sulphur dioxide reduction strategies in winemaking, most studies are based on short-term trials, single vintages, or laboratory-scale fermentations. Moreover, real-time process monitoring is often used primarily as a descriptive tool rather than as an active technological support for fermentation control.

The novelty of the present study lies in the longitudinal validation of a no-added-sulphur-dioxide vinification protocol over four consecutive vintages (2022/2023–2025/2026), combined with the integration of real-time CO_2_ and O_2_ monitoring as a process control strategy. Unlike previous works, this study evaluates the technological feasibility and chemical stability of sulphite-free winemaking under operational winery conditions, explicitly linking fermentation kinetics, gas dynamics, and compositional evolution.

Furthermore, the no-added-SO_2_ protocol is not defined solely by the omission of sulphur dioxide, but by a set of integrated technological and operational measures, including controlled inert gas management, temperature-regulated fermentation, and continuous sensor-based monitoring to enable timely corrective interventions. By demonstrating that stable fermentations and acceptable chemical profiles can be achieved without added sulphites under controlled conditions, this work contributes to a precision oenology framework for sulphur dioxide reduction that complements existing alternative strategies.

## 2. Materials and Methods

### 2.1. Wine Samples and Vinification

Wine samples, produced from *Vitis vinifera* L. cv. Sangiovese grapes, were obtained from the La Cura winery (Massa Marittima, Italy) in four consecutive vintages (2022–2025).

Two vinification protocols were applied in parallel: a conventional protocol involving the addition of sulphur dioxide and a no-added-SO_2_ protocol without the addition of sulphur dioxide.

In both protocols, the grapes were destemmed and crushed, and alcoholic and malolactic fermentation was carried out in temperature-controlled stainless-steel tanks. Selected commercial yeasts and lactic bacteria were inoculated at crushing and at the end of alcoholic fermentation, respectively (Lallemand, Blagnac, France), and fermentation nutrients were added according to the manufacturer’s recommendations. Strict hygiene practices were applied during all stages of vinification.

In the conventional protocol, potassium metabisulphite was added during crushing and racking according to standard oenological practice to ensure antimicrobial and antioxidant protection.

In the no-added-SO_2_ protocol, no sulphur dioxide was added at any stage of vinification. Instead, the protocol was designed as an integrated technological system to compensate for the antimicrobial and antioxidant role typically played by SO_2_.

Specifically, fermentation was carried out in smart tanks equipped with real-time CO_2_ and O_2_ sensors to continuously monitor fermentation dynamics and oxygen availability in the headspace. The temperature was automatically regulated by an internal coil system to maintain optimal conditions for yeast and bacterial activity and avoid thermal stress.

Pumping-over operations were initially performed with air and subsequently with a closed-loop system, without opening the tank. During these operations, inert gas (nitrogen) was introduced to replace oxygen, limit oxidative exposure, and promote controlled cap movement and homogenisation of the fermenting must.

To further support oxidative stability in the absence of added sulphur dioxide, grape seed extracts were added during crushing and racking as alternative antioxidant agents.

The combination of controlled inert gas management, real-time gas monitoring, temperature regulation, strict hygiene practices, use of selected starter cultures, and addition of grape seed extracts was intended to create a selective fermentation environment that would promote rapid yeast dominance, limit oxygen availability, and reduce the risk of microbial deviations in the absence of SO_2_.

The detailed operating conditions were based on the protocol previously described by Mercanti et al. [20], with minor adjustments required by the specific conditions of the vintage.

### 2.2. Smart Tank

**Figure 1:**
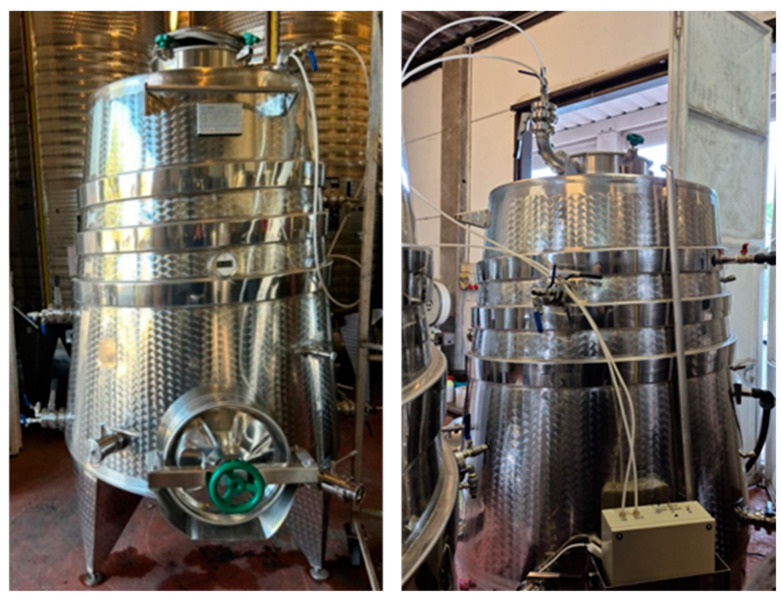
Front view of the tank (**left**), and back view of the tank (**right**).

The experiment was conducted using two truncated cone-shaped smart tanks with a capacity of 15 hL, designed to allow automated and controlled management of the fermentation process. The structure of the tank, made of stainless steel, allows chemical inertia of the material and easy sanitisation, while the truncated cone shape facilitates the collection and subsequent separation of the lees and pomace at the end of fermentation (Figure 1).

The tank is equipped with a temperature control system consisting of an internal steel coil, inside which cold or hot propylene glycol circulates according to the thermal requirements of the process. The coil is connected to an automatic temperature control unit which activates or interrupts the flow of water when the set value is reached. This makes it possible to maintain a constant fermentation temperature, avoiding overheating due to the metabolic activity of the yeasts or sudden temperature changes that could compromise the fermentation kinetics and aromatic quality of the wine.

Another innovative feature of the system is the automatic valves that allow pumping over to be carried out indoors without the need to open the tank. Pumping over is carried out by means of a pump and a circuit of pipes that draw the must from the bottom and return it to the top of the tank, promoting homogenisation of the mass and extraction of phenolic and aromatic compounds from the skins.

During this operation, an inert gas (usually nitrogen or carbon dioxide) is introduced, which exerts controlled pressure inside the tank, causing the cap of pomace to rise and move. The gas is then released through a vent valve.

**Figure 2:**
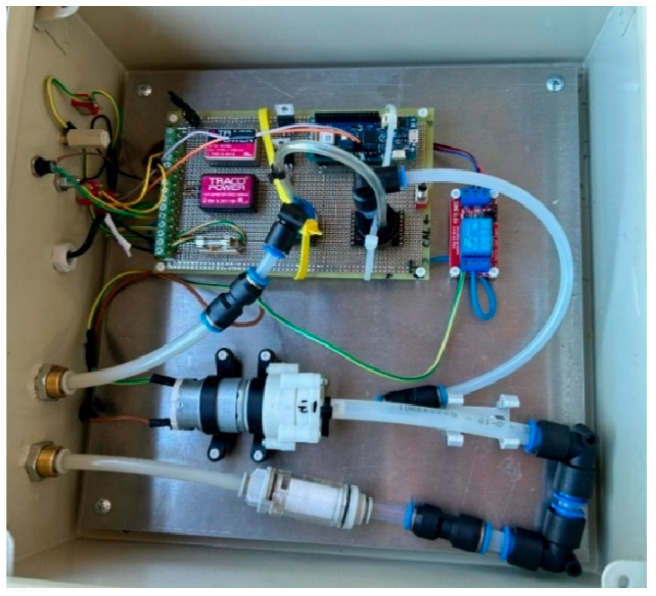
CO_2_ and O_2_ monitoring system.

Furthermore, thanks to the use of this tank, it is possible to monitor the progress of alcoholic fermentation using a sensor system installed directly on the fermentation vat, consisting of two main sensors for the continuous measurement of fermentation gases: CO_2_ and O_2_ present in the headspace of the tank (Figure 2). The data acquired was collected via a digital interface and recorded for real-time analysis of the fermentation process. Sensors were calibrated prior to each fermentation according to the manufacturer’s specifications. Data acquisition and real-time visualization were performed using a dedicated digital interface developed by the research team.

### 2.3. Chemical Analyses

All chemical determinations were performed by the authors at the laboratories of the Department of Agriculture, Food and Environment of the University of Pisa, following the analytical protocols previously described by Mercanti et al. [19], with minor adaptations where indicated.

The sugar content (hexoses, g/L), titratable acidity (expressed in g/L of tartaric acid), pH, L-malic acid (g/L), alcohol content (% *v*/*v*), and net volatile acidity (AVN, g/L of acetic acid) were determined according to the official methods of the International Organisation of Vine and Wine (OIV) [29].

Total phenols (g/L of catechins) were measured using the Folin–Ciocalteu colorimetric test, as previously reported [20], with the following modifications: 1 mL of wine sample (previously diluted 1:10 with deionised water), 5 mL of Folin–Ciocalteu reagent, 15 mL of 20% (*w*/*v*) sodium carbonate, and 79 mL of deionised water were mixed in a 100 mL volumetric flask. After 30 min of incubation at room temperature, the absorbance at 765 nm was measured against a blank. The results were expressed in g/L of catechin equivalents.

Total anthocyanins (g/L malvin) and bleachable anthocyanins (g/L malvin) were evaluated using the hydrochloric acid method and bisulphite bleaching method, respectively, as reported by Aleixandre-Tudo et al. [30].

The free and total sulphur dioxide content was determined using an iCubio iMagic M9 automatic analyser (Shenzhen iCubio Biomedical Technology Co., Ltd., Shenzhen, China), operating in fully automatic mode. The system automatically pipetted the reagents and samples into the cuvette, allowed them to react at a controlled temperature, and measured the absorbance at a specific wavelength. The concentrations of the analytes were calculated using a calibration method [31]. The reagents used were Enzytec™ Liquid SO_2_-free (Code E8610) and Enzytec™ Liquid SO_2_-total (Code E8600). All reagents and standards were purchased from R-Biopharm AG (Darmstadt, Germany).

Each analysis was performed in triplicate.

### 2.4. Statistical Analyses

One-way analysis of variance was performed using CoStat, Version 6.451, CoHort 6.0 Software (Pacific Grove, CA, USA) to assess the presence of significant differences among the investigated samples on the compositional parameters. Regarding analyses, the means were separated by Tukey’s post hoc test using a *p* ≤ 0.05. Each analysis was performed in triplicate.

The composition of the wine samples analysed was also subjected to multivariate statistical analysis using the principal component analysis (PCA) method through the use of the JMP Pro (version 18) statistical package (SAS Institute; Cary, NC, USA).

A two-way ANOVA was also performed to assess the influence of the winemaking protocol, the various vintages and their interaction on the composition of the wine. The presence of a significant influence was assessed using Tukey’s post hoc test with *p* < 0.05.

## 3. Results and Discussion

To comprehensively interpret the differences found in the chemical analyses of wines produced according to the two vinification protocols, a comparison was made between the data for the 2025/2026 vintage and those for previous years (since 2022), taking into account the specific climatic conditions and the choice of harvest period that characterised each year. This approach allowed us to correlate variations in oenological parameters with seasonal weather patterns, offering a broader view of the influence of climate and winemaking protocol on the final characteristics and quality of the wine.

The validation of the proposed innovation required the use of advanced technologies, alongside traditional chemical–analytical assessments, to monitor gas conditions throughout the entire winemaking process. The adoption of this innovative system made it possible to verify the technological feasibility of producing wine without added sulphites, even in harvesting conditions characterised by suboptimal phytosanitary conditions of the grapes. The wines obtained were compared with those produced using traditional methods, evaluating their chemical and compositional characteristics.

### 3.1. Fermentation Kinetics

Analysis of the sugar, alcohol, malic acid, and lactic acid data allows us to evaluate the effectiveness of the two vinification protocols, comparing the speed and completeness of alcoholic and malolactic fermentation and identifying any differences due to the presence or absence of sulphites.

Observation of fermentation parameters during different vintages showed regular trends consistent with those expected for both stages of the winemaking process, both in wines produced using the no-added-SO_2_ protocol and those produced according to the traditional protocol.

**Figure 3:**
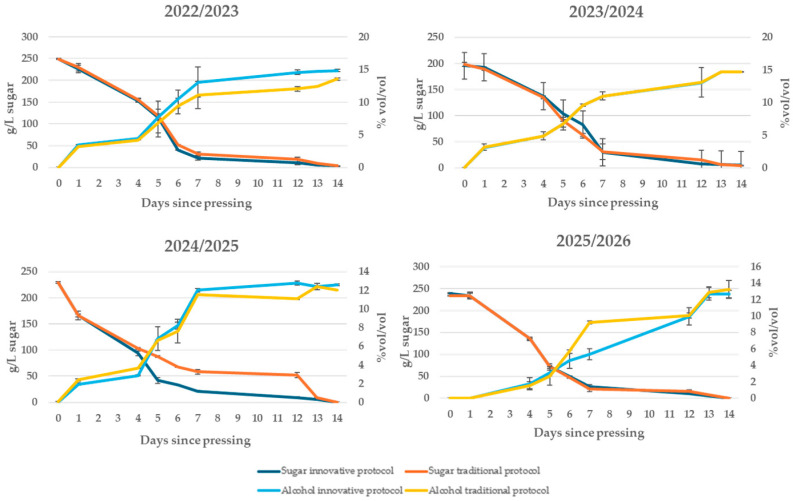
Kinetics of alcoholic fermentation in the first days after pressing in wines produced according to a no-added-SO_2_ protocol and a traditional protocol in four consecutive vintages (2022/2023–2025/2026).

During alcoholic fermentation (Figure 3), a progressive and constant decrease in sugar content was observed until complete depletion, accompanied by a parallel increase in ethanol concentration, a sign of regular and complete fermentation. The fermentation curves show a progressive decrease in sugar concentration and a corresponding increase in ethanol content over time, indicating regular fermentation kinetics in both the innovative and conventional protocols. The absence of arrest or slowdowns confirms that the operating conditions (temperature, oxygenation, availability of nutrients) were optimal for yeast activity, ensuring efficient conversion of sugars into alcohol and carbon dioxide.

**Figure 4:**
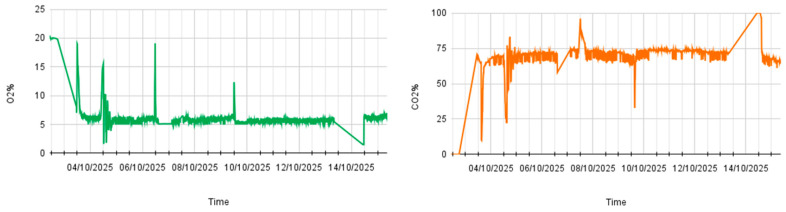
Trend of oxygen and carbon dioxide evolution in the first days after pressing in the smart tank.

In addition to traditional analyses, alcoholic fermentation was also monitored using tailor-made sensors integrated into the smart tank. These devices allowed continuous monitoring of the main process parameters, providing timely information on the evolution of fermentation. The prompt acquisition of data made it possible to identify any changes in operating conditions and intervene during the vinification phase (Figure 4).

The first three vintages (2022/2023–2024/2025) were primarily used for the progressive setup, optimization, and calibration of the sensor-based monitoring system under real winery operating conditions. During these initial phases, sensor configuration, signal stability, and data acquisition protocols were refined to ensure robustness and reproducibility.

The 2025/2026 vintage therefore represents the first fully standardized and technically optimized application of the monitoring system and was selected as a representative example to illustrate the final operational performance of the smart tank and real-time gas monitoring approach.

**Figure 5:**
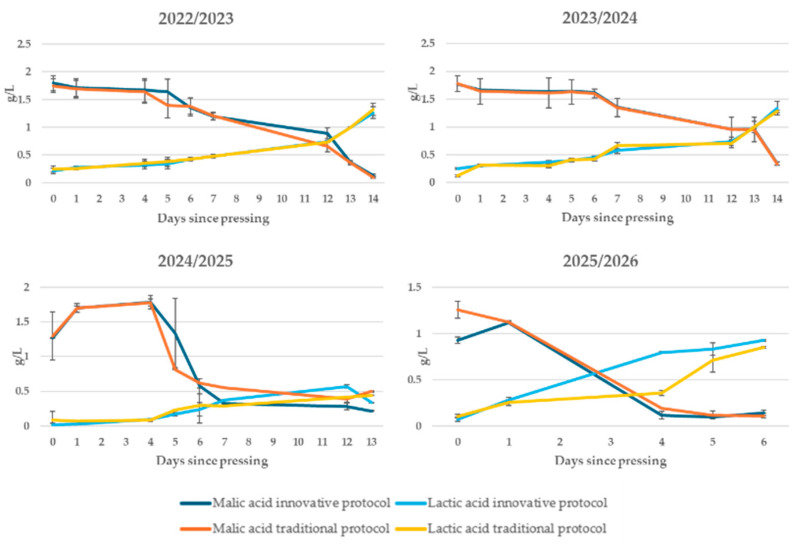
Kinetics of malolactic fermentation in the first days after pressing in wines produced according to a no-added-SO_2_ protocol and a traditional protocol in four consecutive vintages (2022/2023–2025/2026).

Malolactic fermentation (Figure 5) showed an equally regular evolution, with a progressive decrease in malic acid accompanied by a proportional increase in lactic acid, until complete conversion was achieved [32,33]. This behaviour, observed in all tests, confirms the correct activity of lactic bacteria and their ability to adapt to the conditions of the medium even in the absence of added sulphites. The process resulted in the usual reduction in total acidity and a softening of the taste profile, improving the microbiological and sensory stability of the wine.

It is important to note that in all the vintages analysed, both fermentations were completed regularly, without any metabolic stoppages or deviations. Furthermore, despite the absence of added sulphites, the alternative protocol showed a fermentation pattern that was entirely comparable to that observed in the traditional protocol, both in terms of transformation times and substrate conversion efficiency.

### 3.2. Microbiological Control in the Absence of SO_2_

The absence of added sulphur dioxide raises a critical issue regarding microbiological stability, given the well-known antimicrobial role of SO_2_ in suppressing spoilage yeasts and bacteria. In the present study, however, no slow or blocked fermentations were observed, and volatile acidity remained consistently below the sensory perception threshold in all vintages, indicating the absence of significant microbial deviations.

Although no direct analyses of the microbial population were performed, the regular kinetics of alcoholic and malolactic fermentations, the rapid depletion of sugars, the complete conversion of malic acid, and the low volatile acidity values can be interpreted as indirect indicators of effective microbial control.

A plausible mechanistic explanation lies in the rapid predominance of the inoculated Saccharomyces cerevisiae strains, supported by controlled temperature conditions, optimised nutrient availability, and strict hygienic practices. In addition, continuous monitoring of oxygen availability in the headspace and limiting oxygen ingress during racking operations likely contributed to creating a selective environment unfavourable to the growth of spoilage microorganisms.

These results are consistent with previous studies reporting that process control, starter culture dominance and oxygen management can partially compensate for the absence of chemical antimicrobial agents in winemaking [34,35].

### 3.3. Oxidative Management Without Added Sulphur Dioxide

Sulphur dioxide plays a key antioxidant role in winemaking, eliminating oxygen and binding carbonyl compounds such as acetaldehyde. Its absence therefore poses a significant risk of oxidative degradation of aromatic compounds, pigments, and phenolic structures.

In this study, oxidative stability in the absence of added SO_2_ was achieved through an integrated technological approach combining real-time oxygen monitoring, closed-loop pumping operations, controlled inert gas management, temperature regulation, and the use of grape seed extracts with antioxidant activity. The intelligent design of the tank allowed continuous measurement of O_2_ in the headspace and enabled timely corrective interventions to minimise oxygen exposure during critical stages of vinification.

The stability of total and bleachable anthocyanins across different vintages and their comparable evolution between innovative and traditional protocols suggest that no excessive oxidative degradation of pigments occurred under the experimental conditions. Furthermore, the absence of anomalous patterns of SO_2_ consumption and the maintenance of low volatile acidity further support the hypothesis of controlled oxidative conditions. Although specific oxidation markers, such as acetaldehyde, polymeric pigment formation, or browning indices, were not directly measured in this study, combined chemical and sensor-based evidence indicates that oxygen entry was effectively limited. This analytical limitation is acknowledged, and the inclusion of targeted oxidation markers is proposed as a priority for future investigations aimed at further validating the antioxidant robustness of the protocol without the addition of SO_2_.

### 3.4. No-SO_2_ Vinification as Applied Microbial Ecology

Beyond the technological aspects, the no-added-SO_2_ protocol has shown that fermentation outcomes are determined by the design of a selective physical–chemical environment rather than by the use of exogenous chemical stabilisers.

This perspective is consistent with recent studies in other fermentation systems, such as the work by Gao et al. (2025) on tea fermentation, which demonstrated how designed co-culture strategies can actively reshape microbial communities, suppress undesirable microorganisms, and promote beneficial flora to enhance product stability and sensory quality [36].

In the present study, the combination of real-time gas monitoring, controlled oxygen availability, temperature regulation, strict hygiene practices, selected starter cultures, and closed-system operations created a selective fermentation ecosystem that favoured rapid yeast dominance and stable microbial succession in the absence of added sulphur dioxide.

This process control-based approach is in line with broader trends in sustainable fermentation science, where technological precision is used to replace or reduce traditional chemical aids, actively driving microbial dynamics and redox conditions. From this perspective, the SO_2_-free protocol should not be interpreted as a simple omission strategy, but rather as a system-level intervention aimed at managing fermentation ecosystems through precision process control.

### 3.5. Phenol Compounds

The analysis of phenolic compounds, and in particular total phenols, total anthocyanins, and bleachable anthocyanins (Table 1, Table 2 and Table 3), showed a trend mainly driven by vintage-related variability and the phytosanitary status of the grapes at the time of harvest.

During the first two vintages (2022/2023 and 2023/2024), the wines obtained from both the no-added-SO_2_ protocol and the traditional protocol showed high levels of total phenols and anthocyanins, indicative of good phenolic ripeness and excellent grape quality.

In 2022, despite the fact that September was characterised by heavy rainfall, this was well distributed over time, without causing excessive soil saturation or operational difficulties in the vineyard. The regularity of the rainfall, combined with moderate temperatures and good ventilation, allowed the grapes to remain in excellent health, promoting a balance between sugar and phenolic ripeness [37].

The 2023/2024 vintage further consolidated this positive trend, showing the highest values of the entire period for phenols and anthocyanins at the time of harvest. Total phenols reached 3.27 in the no-added-SO_2_ protocol and 3.57 in the traditional protocol, and anthocyanins reached 469.46 ± 0.05 mg/L in the traditional and 361.06 ± 0.05 mg/L in the no-added-SO_2_ protocol. These conditions resulted in wines with high colour intensity, excellent structure, and ageing potential.

Starting in 2024/2025, however, there has been a gradual decline in phenolic values, which was more pronounced in the subsequent 2025/2026 harvest. Total phenols fell on average below 3 g/L, while total and bleachable anthocyanins showed a significant reduction, with values around 100 mg/L for both vinification protocols (at the end of the sampling period, values were 105.39 ± 2.79 for the no-added-SO_2_ protocol and 102.91 ± 0.03 for the traditional protocol).

The 2025 wine has lower overall values than previous vintages, a phenomenon mainly attributable to the compromised phytosanitary state of the grapes. The rains in September 2025, although less abundant than those in 2022, were concentrated in a short period of time, making harvesting difficult and favouring the development of fungal pathogens. This resulted in reduced synthesis and stability of phenolic compounds, with a consequent reduction in the colour intensity and structure of the wine.

However, despite the unfavourable conditions of the 2025 vintage, the analytical values obtained are fully within the commercial standards of the product, guaranteeing a balanced and technically correct wine. Good winemaking management (thanks to the use of a smart tank) has in fact made it possible to preserve part of the residual phenolic and anthocyanin potential, resulting in a wine that is pleasant to drink and has a stable colour, even if less intense than in previous harvests.

### 3.6. Volatile Acidity

Volatile acidity varied depending on the vintage, sampling time, and vinification protocol. In all the vintages considered, the values measured remained well below the sensory perception threshold commonly reported for wine, as stated by OIV (1.2 g/L) [38], indicating no negative effects on the sensory quality of the final product [19].

In the 2022/2023 and 2023/2024 vintages, volatile acidity levels were generally low and comparable between the innovative and traditional vinification, with limited and non-systematic differences. In subsequent vintages (2024/2025 and 2025/2026), an increase in average values was observed, particularly in the no-added-SO_2_ protocol; however, these increases are not sensory or technological in nature, remaining within ranges considered physiological for properly made wines (Table 4).

Overall, the results indicate that the innovative protocol did not lead to a critical increase in volatile acidity, nor did it represent a risk factor for the stability or quality of the wine. On the contrary, the differences observed reflect normal fermentation variations related to the vintage and process conditions, confirming the full compatibility of the no-added-SO_2_ protocol with quality wine production.

### 3.7. Total and Free Sulphur Dioxide

Sulphur dioxide (SO_2_) is the most used additive in vinification since, thanks to its antioxidant potential, it is able to protect the product from various oxidative reactions [39] and also acts as a potent antimicrobial agent [40]. However, due to the numerous adverse effects of this additive, nowadays, consumer preferences are increasingly directed toward the use of natural products, which are considered to be safer and healthier [41,42,43].

In the protocols being compared, SO_2_ management was deliberately different: in traditional vinification, sulphitation was carried out, while in the innovative protocol, no SO_2_ was added; therefore, the SO_2_ detected in the innovative samples is attributable to endogenous production by yeasts and subsequent equilibrium with the oxidisable compounds in the must/wine.

**Figure 6:**
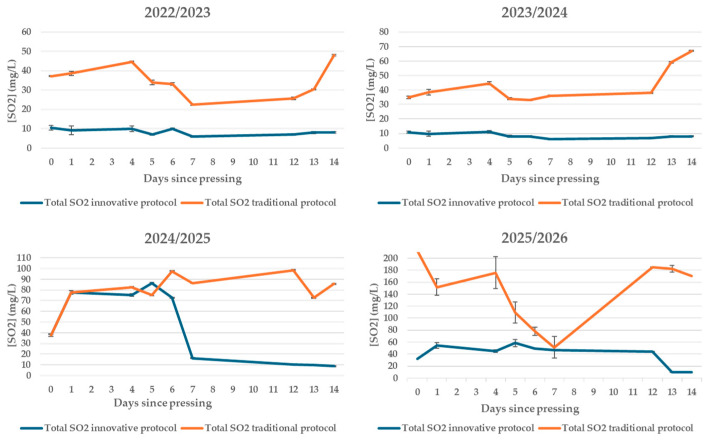
Trend of total sulphur dioxide in the first days after pressing in wines produced according to a no-added-SO_2_ protocol and a traditional protocol in four consecutive vintages (2022/2023–2025/2026).

In all vintages, the total SO_2_ in the traditional protocol was higher overall than in the innovative protocol, consistent with the use of metabisulphite. In the innovative protocol, the lower and relatively stable levels support the technological objective of obtaining a wine without added sulphites while maintaining a pattern compatible with regular fermentation (Figure 6).

In the 2025/2026 vintage, the traditional profile clearly shows a corrective intervention: after the initial consumption phase, there was a net increase in total SO_2_ (approximately halfway through the observation period), consistent with the addition of sulphites to restore product protection. The absence of similar increases in the innovative protocol is consistent with the “no-added-sulphites” protocol and with the fact that the SO_2_ measured derives exclusively from yeast metabolism [44].

The trend in free SO_2_ in the first 14 days after crushing confirms the clear and systematic differences between the no-added-SO_2_ protocol and traditional vinification, consistent with the different sulphur management strategies. In the traditional protocol, free SO_2_ levels were consistently higher. Conversely, in the no-added-SO_2_ protocol, where no SO_2_ was added, the values observed are attributable exclusively to the endogenous production of yeasts and to the dynamics of binding and consumption during fermentation.

**Figure 7:**
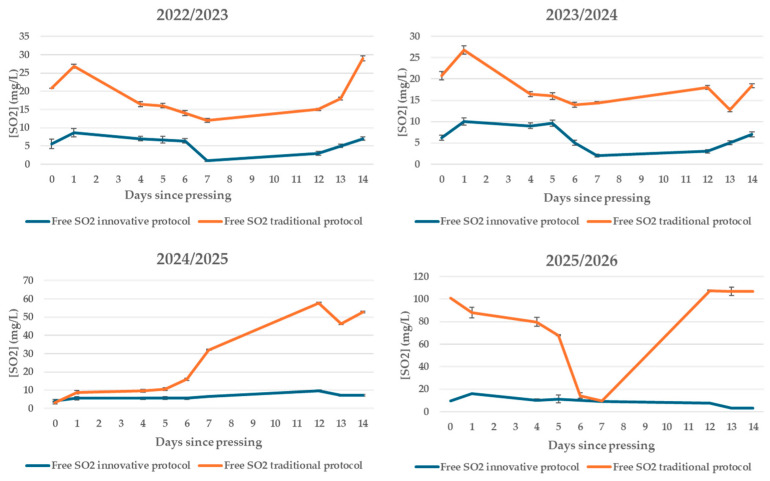
Trend of free sulphur dioxide in the first days after pressing in wines produced according to a no-added-SO_2_ protocol and a traditional protocol in four consecutive vintages (2022/2023–2025/2026).

In all the vintages considered, free SO_2_ in the no-added-SO_2_ protocol remained at low and relatively stable values, without showing sudden increases, confirming the consistency of the “no-added-sulphites” protocol. In the traditional protocol, in contrast, the trend in free SO_2_ reflects technological interventions aimed at restoring antioxidant and antimicrobial protection levels, particularly in more recent vintages (Figure 7).

The trend in free SO_2_ is consistent with that observed for total SO_2_ and volatile acidity. In particular, in the innovative protocol, low levels of free SO_2_ were not associated with abnormal increases in volatile acidity, which always remained below the sensory perception threshold. This result indicates that the absence of added sulphites did not compromise the stability of the fermentation process under the experimental conditions considered.

### 3.8. Principal Component Analyses (PCA)

**Figure 8:**
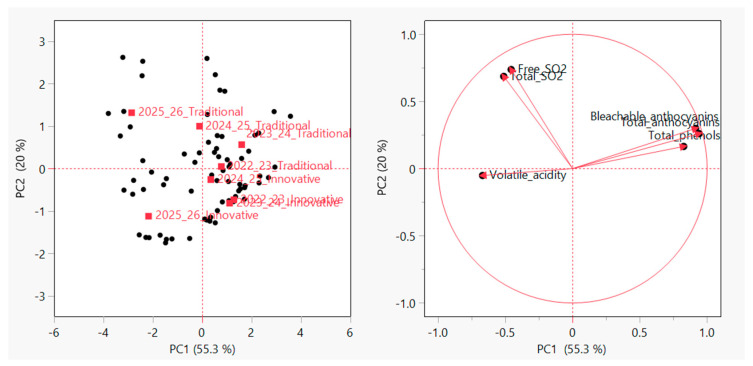
Principalcomponent analysis (PCA) score and loading plots based on chemical parameters of wines produced using innovative and traditional vinification protocols across four consecutive vintages (2022/2023–2025/2026).

Principal component analysis (PCA) was applied as an exploratory multivariate tool to integrate the information provided by individual chemical parameters and to assess the relative contribution of the vintage and vinification protocol to the overall variability of the wines. The first two principal components accounted for 75.3% of the total variance, indicating a satisfactory representation of the data set and allowing for a meaningful interpretation of the multivariate structure. The first principal component (PC1) was mainly associated with total phenols, total anthocyanins, and bleachable anthocyanins. This result highlights the dominant role of vintage-related factors on the phenolic composition of wines, in agreement with the results of the two-way ANOVA and with the well-known influence of climatic conditions and grape maturity on phenolic extraction and stability (Table 5). The distribution of samples along PC1 confirms that phenolic variability is determined mainly by the raw material rather than the vinification protocol applied. The second principal component (PC2) was determined by variables related to sulphur dioxide, with volatile acidity contributing in the opposite direction (Figure 8). This component reflects the differences in sulphur dioxide management between the innovative and traditional protocols. Wines produced using the traditional protocol tended to score higher along PC2, in line with sulphite additions, while innovative wines clustered in regions characterised by lower SO_2_ levels, reflecting the absence of added sulphites and the exclusive contribution of SO_2_ derived from yeast. It is important to note that innovative wines showed a stable chemical profile despite the absence of sulphite additions. Furthermore, the opposite orientation of volatile acidity with respect to sulphur dioxide variables, combined with its low absolute values, suggests that the reduced SO_2_ levels in the no-added-SO_2_ protocol did not lead to increased fermentative or microbiological instability. Overall, PCA supports the conclusion that vintage is the main source of chemical variability, while sulphur dioxide management acts as a secondary but clearly identifiable factor. Multivariate analysis clearly demonstrated that vintage was the dominant factor influencing wine composition, particularly phenolic and anthocyanin profiles, whereas the vinification protocol (innovative vs. conventional) represented a secondary but still identifiable source of variability.

This finding highlights that climatic and seasonal conditions exert a stronger influence on wine composition than sulphur dioxide management strategies and that any technological intervention aimed at reducing SO_2_ must be interpreted within the broader context of vintage-dependent variability.

Accordingly, the effect of the no-added-SO_2_ protocol should be regarded as conditional and process-dependent, rather than as a primary driver of compositional change.

## 4. Conclusions

This work has demonstrated that sulphur dioxide-free winemaking is technically feasible under controlled cellar conditions, if supported by integrated process control.

Over four consecutive vintages, the no-added-SO_2_ protocol consistently produced wines with very low levels of total and free SO_2_, with total SO_2_ ranging from 10 to 58 mg/L and free SO_2_ from 0 to 1 mg/L, attributable exclusively to endogenous yeast production, while maintaining regular alcoholic and malolactic fermentation and volatile acidity values below the sensory threshold (1.2 g/L).

Alcoholic fermentation proceeded regularly in all trials, with fermentation completed within approximately 13–15 days in all vintages, and no technological deviations or fermentation arrests were observed.

The oxidative and microbiological risks typically mitigated by SO_2_ were managed through a combined strategy that included closed-loop operations, controlled use of inert gas, real-time CO_2_/O_2_ monitoring, temperature regulation, strict hygiene, and the addition of grape seed extracts as alternative antioxidant agents.

Multivariate analysis confirmed that vintage was the dominant factor influencing most compositional parameters, particularly phenolic and anthocyanin profiles, while sulphur dioxide management represented a secondary but clearly identifiable source of variability.

Overall, the no-added-SO_2_ protocol should be considered a complementary tool for SO_2_ reduction rather than a universal substitute for conventional sulphite management. Its successful application remains subject to precise technological control to compensate for the antioxidant and antimicrobial functions normally provided by sulphur dioxide.

Future perspectives will focus on validating the proposed no-added-SO_2_ protocol on different grape varieties to assess its broader applicability.

## Figures and Tables

**Table 1:** Trend in total phenols (g/L catechins) in the first days after pressing in wines produced according to a no-added-SO_2_ protocol and a traditional protocol in four consecutive vintages (2022/2023–2025/2026).

	Year 2022/2023		Year 2023/2024		Year 2024/2025		Year 2025/2026	
Days Since Pressing	Innovative Vinification	Traditional Vinification	Innovative Vinification	Traditional Vinification	Innovative Vinification	Traditional Vinification	Innovative Vinification	Traditional Vinification
0	1.17 ± 0.03 ^B^	1.72 ± 0.02 ^A^	3.27 ± 0.00 ^B^	3.57 ± 0.00 ^A^	1.05 ± 0.09 ^A^	1.06 ± 0.03 ^A^	1.00 ± 0.01 ^B^	1.15 ± 0.02 ^A^
1	2.87 ± 0.01 ^B^	3.10 ± 0.03 ^A^	3.30 ± 0.02 ^B^	3.60 ± 0.02 ^A^	1.50 ± 0.02 ^A^	1.26 ± 0.05 ^B^	1.37 ± 0.01 ^A^	1.25 ± 0.07 ^B^
4	3.96 ± 0.02 ^A^	3.58 ± 0.02 ^B^	3.35 ± 0.00 ^B^	3.69 ± 0.00 ^A^	2.41 ± 0.00 ^A^	2.14 ± 0.45 ^B^	1.53 ± 0.12 ^B^	1.66 ± 0.02 ^A^
5	3.80 ± 0.00 ^A^	3.78 ± 0.00 ^B^	3.20 ± 0.00 ^B^	3.49 ± 0.00 ^A^	3.42 ± 0.27 ^A^	3.21 ± 0.28 ^B^	1.98 ± 0.01 ^B^	2.06 ± 0.00 ^A^
6	3.64 ± 0.00 ^A^	3.64 ± 0.01 ^A^	3.05 ± 0.00 ^B^	3.29 ± 0.00 ^A^	2.89 ± 0.03 ^A^	2.82 ± 0.00 ^B^	2.27 ± 0.00 ^B^	2.31 ± 0.00 ^A^
7	3.33 ± 0.06 ^A^	3.18 ± 0.01 ^B^	3.02 ± 0.00 ^B^	3.41 ± 0.00 ^A^	3.10 ± 0.02 ^B^	3.14 ± 0.02 ^A^	2.28 ± 0.05 ^B^	2.34 ± 0.03 ^A^
12	3.02 ± 0.11 ^A^	2.72 ± 0.00 ^B^	2.98 ± 0.04 ^B^	3.90 ± 0.06 ^A^	2.80 ± 0.09 ^A^	2.52 ± 0.00 ^B^	2.31 ± 0.01 ^B^	2.43 ± 0.03 ^A^
13	2.89 ± 0.17 ^A^	2.70 ± 0.13 ^B^	3.03 ± 0.00 ^B^	3.40 ± 0.02 ^A^	2.55 ± 0.02 ^B^	2.70 ± 0.34 ^A^	2.46 ± 0.01 ^B^	2.49 ± 0.05 ^A^
14	2.56 ± 0.20 ^A^	2.54 ± 0.09 ^A^	3.12 ± 0.01 ^B^	3.45 ± 0.01 ^A^	2.66 ± 0.01 ^A^	2.53 ± 0.01 ^B^	2.61 ± 0.01 ^A^	2.55 ± 0.05 ^B^

Values expressed as mean ± standard deviation. Different letters in the same row and year indicate statistically significant differences between innovative and traditional vinification systems (*p* < 0.05).

**Table 2:** Trend in total anthocyanins (mg/L malvin) in the first days after pressing in wines produced according to a no-added-SO_2_ protocol and a traditional protocol in four consecutive vintages (2022/2023–2025/2026).

	Year 2022/2023		Year 2023/2024		Year 2024/2025		Year 2025/2026	
Days Since Pressing	Innovative Vinification	Traditional Vinification	Innovative Vinification	Traditional Vinification	Innovative Vinification	Traditional Vinification	Innovative Vinification	Traditional Vinification
0	139.65 ± 1.88 ^A^	70.49 ± 0.94 ^B^	361.06 ± 0.05 ^B^	469.46 ± 0.05 ^A^	25.84 ± 0.89 ^B^	27.80 ± 0.09 ^A^	5.49 ± 1.55 ^A^	5.49 ± 1.55 ^A^
1	193.85 ± 0.47 ^B^	230.42 ± 1.41 ^A^	324.72 ± 1.60 ^B^	473.58 ± 0.05 ^A^	117.71 ± 0.75 ^A^	112.62 ± 2.68 ^B^	39.27 ± 4.28 ^B^	43.42 ± 0.66 ^A^
4	364.42 ± 2.82 ^A^	356.44 ± 1.90 ^B^	301.91 ± 0.00 ^B^	415.16 ± 0.09 ^A^	224.04 ± 0.85 ^A^	221.74 ± 0.33 ^B^	60.91 ± 4.23 ^A^	49.82 ± 0.58 ^B^
5	307.33 ± 0.14 ^A^	286.58 ± 0.33 ^B^	253.40 ± 0.09 ^B^	351.65 ± 0.14 ^A^	247.91 ± 2.82 ^B^	261.15 ± 1.03 ^A^	62.04 ± 0.33 ^A^	56.23 ± 0.52 ^B^
6	297.85 ± 3.53 ^A^	273.45 ± 0.19 ^B^	204.89 ± 0.19 ^B^	288.14 ± 0.19 ^A^	260.12 ± 0.85 ^A^	250.31 ± 2.26 ^B^	69.26 ± 5.78 ^B^	78.17 ± 1.13 ^A^
7	288.00 ± 5.60 ^A^	268.57 ± 0.20 ^B^	206.45 ± 0.33 ^B^	289.47 ± 0.38 ^A^	291.94 ± 4.98 ^B^	312.92 ± 2.59 ^A^	77.11 ± 3.71 ^B^	87.45 ± 0.05 ^A^
12	286.50 ± 7.80 ^A^	265.23 ± 0.32 ^B^	153.35 ± 3.39 ^B^	226.20 ± 9.92 ^A^	308.16 ± 3.39 ^B^	310.32 ± 11.99 ^A^	88.26 ± 1.32 ^B^	89.11 ± 15.05 ^A^
13	285.00 ± 10.00 ^A^	263.70 ± 5.16 ^B^	184.80 ± 1.50 ^B^	255.19 ± 1.27 ^A^	309.42 ± 7.99 ^B^	320.30 ± 10.30 ^A^	93.07 ± 2.52 ^B^	93.20 ± 2.49 ^A^
14	285.00 ± 10.00 ^A^	263.00 ± 6.23 ^B^	164.65 ± 3.39 ^B^	230.69 ± 11.76 ^A^	299.82 ± 5.69 ^B^	321.89 ± 2.21 ^A^	105.39 ± 2.79 ^A^	102.91 ± 0.03 ^B^

Values expressed as mean ± standard deviation. Different letters in the same row and year indicate statistically significant differences between innovative and traditional vinification systems (*p* < 0.05).

**Table 3:** Trend in bleachable anthocyanins (mg/L malvin) in the first days after pressing in wines produced according to a no-added-SO_2_ protocol and a traditional protocol in four consecutive vintages (2022/2023–2025/2026).

	Year 2022/2023		Year 2023/2024		Year 2024/2025		Year 2025/2026	
Days Since Pressing	Innovative Vinification	Traditional Vinification	Innovative Vinification	Traditional Vinification	Innovative Vinification	Traditional Vinification	Innovative Vinification	Traditional Vinification
0	53 ± 0.62 ^B^	135 ± 19.80 ^A^	246.23 ± 0.25 ^B^	354.99 ± 0.00 ^A^	10.68 ± 1.11 ^A^	8.31 ± 1.61 ^B^	2.80 ± 3.59 ^B^	3.06 ± 0.99 ^A^
1	61 ± 4.95 ^B^	172 ± 9.90 ^A^	216.69 ± 0.06 ^B^	332.63 ± 0.19 ^A^	45.33 ± 0.87 ^B^	81.81 ± 3.84 ^A^	16.10 ± 1.61 ^A^	14.96 ± 1.24 ^B^
4	303 ± 3.71 ^A^	273 ± 7.42 ^B^	193.99 ± 0.12 ^B^	241.46 ± 0.93 ^A^	114.54 ± 1.11 ^B^	127.09 ± 6.13 ^A^	19.29 ± 2.04 ^B^	26.12 ± 0.80 ^A^
5	250 ± 1.98 ^A^	199 ± 0.37 ^B^	183.97 ± 0.12 ^B^	230.93 ± 0.53 ^A^	170.76 ± 7.24 ^B^	201.29 ± 1.18 ^A^	23.01 ± 1.61 ^B^	31.02 ± 1.79 ^A^
6	243 ± 0.00 ^A^	190 ± 0.00 ^B^	173.95 ± 0.12 ^B^	220.41 ± 0.12 ^A^	183.27 ± 6.99 ^B^	218.05 ± 0.49 ^A^	23.84 ± 4.27 ^B^	39.38 ± 2.72 ^A^
7	227 ± 2.00 ^A^	187 ± 0.00 ^B^	102.99 ± 0.12 ^B^	132.21 ± 0.37 ^A^	245.09 ± 6.31 ^B^	280.79 ± 1.86 ^A^	42.09 ± 2.85 ^B^	53.24 ± 2.91 ^A^
12	210 ± 4.10 ^A^	183 ± 0.00 ^B^	149.41 ± 26.67 ^B^	191.67 ± 12.19 ^A^	266.83 ± 1.79 ^B^	286.52 ± 3.65 ^A^	52.19 ± 2.91 ^B^	52.72 ± 2.82 ^A^
13	206 ± 6.70 ^A^	175 ± 3.54 ^B^	131.91 ± 0.19 ^B^	169.53 ± 0.31 ^A^	262.81 ± 0.43 ^B^	278.43 ± 1.11 ^A^	61.25 ± 4.95 ^A^	58.65 ± 3.12 ^B^
14	201 ± 4.54 ^A^	150 ± 0.98 ^B^	111.30 ± 7.30 ^B^	161.83 ± 0.68 ^A^	255.46 ± 2.78 ^B^	263.81 ± 2.47 ^A^	81.81 ± 3.84 ^A^	79.80 ± 3.46 ^B^

Values expressed as mean ± standard deviation. Different letters in the same row and year indicate statistically significant differences between innovative and traditional vinification systems (*p* < 0.05).

**Table 4:** Trend in volatile acidity (g/L of acetic) in the first days after pressing in wines produced according to a no-added-SO_2_ protocol and a traditional protocol in four consecutive vintages (2022/2023–2025/2026).

	Year 2022/2023		Year 2023/2024		Year 2024/2025		Year 2025/2026	
Days Since Pressing	Innovative Vinification	Traditional Vinification	Innovative Vinification	Traditional Vinification	Innovative Vinification	Traditional Vinification	Innovative Vinification	Traditional Vinification
0	0.09 ± 0.01 ᴬ	0.09 ± 0.01 ᴮ	0.10 ± 0.01 ᴬ	0.09 ± 0.01 ᴮ	0.39 ± 0.03 ᴬ	0.34 ± 0.03 ᴮ	0.41 ± 0.00 ᴬ	0.41 ± 0.00 ᴬ
1	0.30 ± 0.07 ᴬ	0.29 ± 0.08 ᴮ	0.33 ± 0.04 ᴬ	0.28 ± 0.10 ᴮ	0.29 ± 0.06 ᴬ	0.25 ± 0.07 ᴮ	0.65 ± 0.01 ᴬ	0.62 ± 0.36 ᴮ
4	0.27 ± 0.02 ᴮ	0.27 ± 0.02 ᴬ	0.27 ± 0.02 ᴮ	0.27 ± 0.01ᴬ	0.33 ± 0.06 ᴮ	0.42 ± 0.06 ᴬ	0.62 ± 0.01 ᴬ	0.51 ± 0.02 ᴮ
5	0.29 ± 0.01 ᴮ	0.29 ± 0.01 ᴬ	0.28 ± 0.00 ᴮ	0.29 ± 0.01ᴬ	0.32 ± 0.05 ᴬ	0.31 ± 0.09 ᴮ	0.68 ± 0.00 ᴬ	0.59 ± 0.00 ᴮ
6	0.43 ± 0.06 ᴬ	0.42 ± 0.07 ᴮ	0.45 ± 0.04 ᴬ	0.42 ± 0.08 ᴮ	0.31 ± 0.07 ᴮ	0.37 ± 0.09 ᴬ	0.65 ± 0.00 ᴬ	0.62 ± 0.36 ᴮ
7	0.36 ± 0.00 ᴮ	0.36 ± 0.00 ᴬ	0.35 ± 0.04 ᴮ	0.37 ± 0.06 ᴬ	0.36 ± 0.06 ᴮ	0.39 ± 0.13 ᴬ	0.64 ± 0.00 ᴬ	0.59 ± 0.00 ᴮ
12	0.41 ± 0.01 ᴬ	0.41 ± 0.01 ᴮ	0.42 ± 0.03 ᴬ	0.40 ± 0.00 ᴮ	0.38 ± 0.07 ᴮ	0.40 ± 0.15 ᴬ	0.62 ± 0.02 ᴮ	0.66 ± 0.00 ᴬ
13	0.43 ± 0.02 ᴮ	0.44 ± 0.02 ᴬ	0.43 ± 0.04 ᴮ	0.44 ± 0.01 ᴬ	0.32 ± 0.05 ᴮ	0.37 ± 0.11 ᴬ	0.71 ± 0.02 ᴬ	0.48 ± 0.04 ᴮ
14	0.45 ± 0.02 ᴬ	0.44 ± 0.02 ᴮ	0.45 ± 0.03 ᴬ	0.44 ± 0.01 ᴮ	0.35 ± 0.06 ᴬ	0.31 ± 0.13 ᴮ	0.71 ± 0.00 ᴬ	0.50 ± 0.04 ᴮ

Values expressed as mean ± standard deviation. Different letters in the same row and year indicate statistically significant differences between innovative and traditional vinification systems (*p* < 0.05).

**Table 5:** Two-way ANOVA was performed to assess the influence of vinification protocol and vintages.

Parameters	Vinification Protocol (WP)	Year (Y)	WP × Y
Total phenols (g/L catechins)	n.s.	***	n.s.
Total anthocyanins (mg/L malvin)	n.s.	***	n.s.
Bleachable anthocyanins (mg/L malvin)	n.s.	***	n.s.
Volatile acidity (g/L of acetic acid)	n.s.	***	n.s.
Total sulphur dioxide (mg/L)	***	***	n.s.
Free sulphur dioxide (mg/L)	***	***	***

*** *p* < 0.0001; n.s. not significant.

## Data Availability

The original contributions presented in the study are included in the article. Further inquiries can be directed to the corresponding author.
